# Toward an Understanding of Healthy Cognitive Aging: The Importance of Lifestyle in Cognitive Reserve and the Scaffolding Theory of Aging and Cognition

**DOI:** 10.1093/geronb/gbac197

**Published:** 2022-12-21

**Authors:** Elise J Oosterhuis, Kate Slade, Patrick J C May, Helen E Nuttall

**Affiliations:** Department of Psychology, Lancaster University, Lancaster, UK; Department of Psychology, Lancaster University, Lancaster, UK; Department of Psychology, Lancaster University, Lancaster, UK; Department of Psychology, Lancaster University, Lancaster, UK

**Keywords:** Compensatory mechanisms, Intervention, Neurodegenerative disease, Shortcomings

## Abstract

The World Health Organization (WHO) aims to improve our understanding of the factors that promote healthy cognitive aging and combat dementia. Aging theories that consider individual aging trajectories are of paramount importance to meet the WHO’s aim. Both the revised Scaffolding Theory of Aging and Cognition (STAC-r) and Cognitive Reserve theory (CR) offer theoretical frameworks for the mechanisms of cognitive aging and the positive influence of an engaged lifestyle. STAC-r additionally considers adverse factors, such as depression. The two theories explain different though partly overlapping aspects of cognitive aging. Currently, it is unclear where the theories agree and differ and what compensation mechanism of age-related cognitive decline might be better explained by either STAC-r, CR, or by both. This review provides an essential discussion of the similarities and differences between these prominent cognitive aging theories, their implications for intervention methods and neurodegenerative disease, and significant shortcomings that have not yet been addressed. This review will direct researchers to common insights in the field and to intervention targets and testable hypotheses for future research. Future research should investigate the potential use of STAC-r in neurodegenerative diseases and provide clarity as to what combination of factors build CR, including their relative importance and when in life they are most effective.

## Examining the Mechanisms of Cognitive Decline

The global population over age 65 is expected to increase by 120% between 2019 and 2050 (United Nations et al., 2020). With a rapidly aging population, dementia also becomes more prevalent. Mild cognitive changes are an inevitable part of healthy aging. However, declines in cognitive function beyond the expected age-related changes may signal a transition into dementia. The World Health Organization emphasizes the importance of understanding mechanisms of healthy aging to create evidence-based intervention and prevention strategies to combat dementia (World Health Organization, 2020). Therefore, it is crucial to investigate what factors promote healthy cognitive aging and slow down the transition to dementia.

Approximately 40% of dementia cases can be delayed or prevented through a healthy lifestyle that reduces risk factors, such as physical inactivity, low education, and social isolation (Livingston et al., 2020). Such risk factors contribute to heterogenous aging trajectories, in which some people age better than others (Daskalopoulou et al., 2019). Why certain subsections of the population, such as centenarians, are able to maintain cognitive functioning at very old age (e.g., Beker et al., 2021) is unclear. Hence, there is a need for aging theories that consider individual trajectories of cognitive aging.

Currently, there are several aging theories that demonstrate some overlap in their constructs, such as changes in brain activity patterns with age (for a review, see Barulli & Stern, 2013). Attempts have been made to form a consensus of terminology and constructs (Cabeza et al., 2018; Stern et al., 2020). Previous reviews have mainly focused on finding common ground between different cognitive aging theories (Barulli & Stern, 2013). However, determining both similarities and differences between theories of cognitive aging will allow us to identify what cognitive aging mechanisms are agreed upon and what mechanisms need clarification.

Several cognitive aging theories, such as the hemispheric asymmetry reduction in older adults (HAROLD; Cabeza, 2002; see Table 1 for an overview of these theories), aim to explain the mechanisms underlying cognitive decline. However, they might not be comprehensive enough to fully explain the complex mechanism involved in cognitive aging (Festini et al., 2018). In contrast, the revised Scaffolding Theory of Aging and Cognition (STAC-r; Reuter-Lorenz & Park, 2014) and the theory of Cognitive Reserve (CR; Stern et al., 2020) present important viewpoints on the underpinnings of healthy cognitive aging by taking a multifaceted approach. The theories have both similarities and important differences. This review aims to evaluate which aspects of cognitive aging might be explained best by either theory. We will focus on the effect of lifestyle factors and brain health on cognitive decline and discuss how the two theories could serve as models in predicting dementia. Through critical comparison, we will direct researchers to common insights in the field and to intervention targets and testable hypotheses for future research. We first review current theories of cognitive aging and contrast these theories with CR and STAC-r. We then discuss the similarities and differences between CR and STAC-r, and their implications for intervention methods and neurodegenerative disease. Finally, for both theories, we identify significant shortcomings that have not yet been addressed and provide directions for future research.

**Table 1.** Description of the Main Theories on Age-Related Cognitive Decline

Cognitive Aging Theory	Description
Compensation-related utilization of neural circuits hypothesis (Reuter-Lorenz & Cappell, 2008)	• As task demands increase, the brain recruits more neural resources to maintain task performance • When task demands are too high, the brain is not able to effectively compensate for the increase in load (i.e., crunch point) • Older adults experience this crunch point sooner than younger adults and age-related decreases in cognitive task performance become apparent • Compensatory processes benefit from, for example, cognitive training and exercise, and are influenced negatively by genetic disadvantages
Hemispheric asymmetry reduction in older adults (Cabeza, 2002)	• Older adults recruit bilateral prefrontal cortices during cognitive tasks to maintain task performance • Younger adults show more unilateral brain activity
Posterior–anterior shift in aging (Davis et al., 2008)	• Older adults maintain optimal task performance despite brain activity decreases in posterior brain regions due to brain activity increases in anterior regions
Neural dedifferentiation hypothesis (Li et al., 2001)	• With age, brain regions and networks become less functionally specific to certain perceptual inputs or cognitive processes • Brain activation will become more widespread in older compared to younger adults

## Theories of Age-Related Cognitive Decline

Cognitive aging theories, such as the compensation-related utilization of neural circuits hypothesis (CRUNCH; Reuter-Lorenz & Cappell, 2008), HAROLD (Cabeza, 2002), and posterior–anterior shift in aging (PASA; Davis et al., 2008), explain age-related changes in cognitive functioning through modulations in brain activation patterns. However, HAROLD and PASA might not explain why some individuals are better able to compensate for age-related cognitive decline than others. A strong point of CRUNCH is that it considers the effect of external factors, such as exercise or genetic disadvantages, on successful compensation. A weakness is that CRUNCH may not be applicable to every cognitive domain, such as visuospatial working memory, as the crunch point cannot always be found in older adults despite high task loads and increases in brain activity (e.g., Jamadar, 2020). In addition, HAROLD proposes that age-related changes in brain activity may reflect neural dedifferentiation, wherein brain activation patterns become less specific (Cabeza, 2002; Li et al., 2001). However, the relationship between cognition and neural dedifferentiation can also be demonstrated in younger adults (see Koen & Rugg, 2019). Hence, it can be hypothesized that neural dedifferentiation might not underlie age-related declines in all cognitive domains. Moreover, the effects of lifestyle choices on neural dedifferentiation are not accounted for and are currently unknown (Koen & Rugg, 2019).

**Figure 1.**
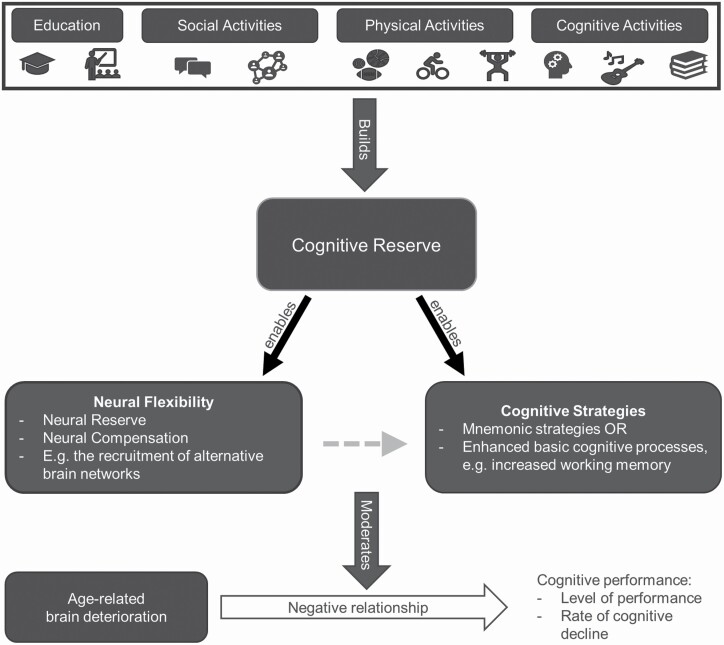
A schematic representation of Cognitive Reserve theory. Cognitive Reserve enables Neural Flexibility and Cognitive Strategies, which both moderate the negative influence of age-related brain deterioration on cognitive performance. The gray dashed arrow indicates that the neuronal processes inevitably influence cognitive processes.

External factors could explain why some individuals are better able to compensate for age- and disease-related cognitive difficulties than others. CR (Stern et al., 2020) states that cognitive “reserve” is accumulated through lifestyle choices and lifetime experiences, such as social activities and education (Figure 1). To measure CR, researchers use individual or combinations of proxies, including years of education, IQ, and engagement in leisure, social, or physical activities. The accumulated CR increases cognitive and neural flexibility by enabling the use of cognitive strategies, strengthening existing brain networks, or recruiting alternative brain networks. To illustrate, if two individuals have similar age-related structural brain changes, one individual could still be able to maintain cognitive performance due to higher CR levels while the performance of the other individual falters. The person who maintains cognitive performance has higher CR levels that enable the use of alternative cognitive strategies and functional brain networks. Stern and colleagues hypothesized that CR proxies predict both cognitive performance and the rate of cognitive decline, and that high CR slows down disease-related cognitive decline and delays the onset of dementia (Stern et al., 2020).

An important limitation of CR is that, despite attempts and the use of neural proxies (e.g., blood flow), no explicit neural basis has been proposed. Instead, brain reserve and brain maintenance are often discussed in conjunction with cognitive reserve (Barulli & Stern, 2013). Brain reserve reflects the brain’s anatomical resources (e.g., the number of neurons) and is fixed because it cannot be altered by life experiences (Stern et al., 2020). Individuals with more brain reserve would be better able to cope with pathology, delaying the manifestation of cognitive changes. Quantitative measurements of brain structure, which reflect brain reserve, can predict when cognitive disfunction occurs. Although brain reserve and cognitive reserve are discussed as separate concepts, Cabeza and colleagues (2018) argued that the concepts should be merged under the umbrella term “reserve” because cognition is based in the brain. Brain maintenance refers to the mechanisms of repair and plasticity that can protect against structural decline of the brain. Lifestyle choices and genetics feed into brain maintenance, and can delay the development of brain pathology and increase the brain reserve threshold for cognitive decline. To summarize, brain reserve protects against the effects of brain pathology but cannot be altered by life experiences. Brain maintenance protects the brain against the development of pathology and is modulated through genetics and life experiences. Finally, CR explains individual differences in the ability to cope with cognitive difficulties resulting from brain pathology through differences in life experiences. Recently, researchers started to use the umbrella term of “resilience” to describe the ameliorating effect of CR, brain reserve, and brain maintenance on slowing down age-related brain changes and pathology (Stern et al., 2020).

**Figure 2.**
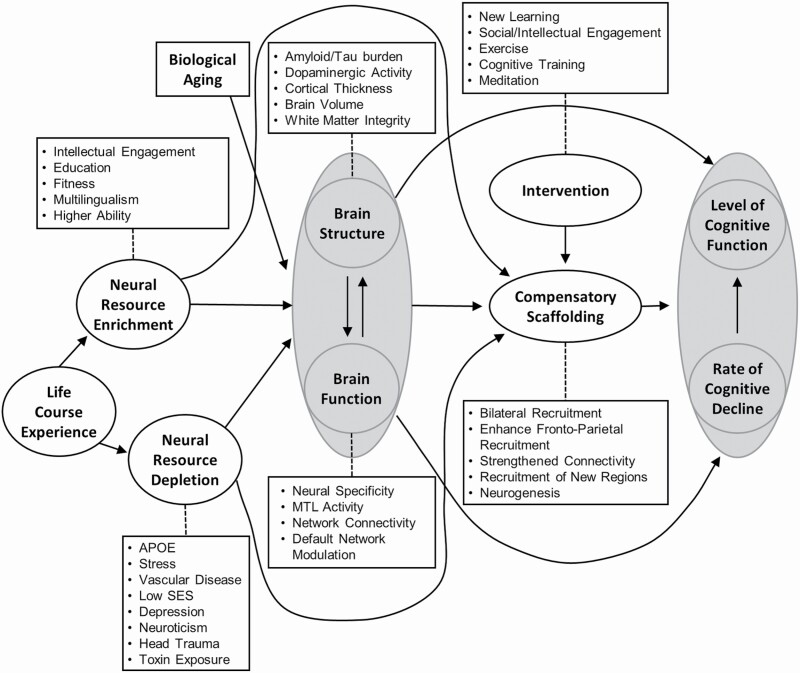
A conceptual model of the Scaffolding Theory of Aging and Cognition—revised. The arrows indicate the influence of one model’s component over another. Adapted from Reuter-Lorenz and Park (2014).

However, the different concepts of reserve have not yet been integrated into a single model. A comprehensive model is needed where such concepts are linked clearly to make specific predictions about the complex mechanisms of cognitive aging. Moreover, whilst CR does predict lower cognition and faster cognitive decline in people with low CR, it does not include adverse factors, such as depression. STAC-r offers a multifaceted framework that includes adverse life factors (Figure 2; Reuter-Lorenz & Park, 2014). According to STAC-r, cognitive decline is caused by age-related deterioration of brain structure and function, such as a reduction in brain volume and functional brain connectivity. “Scaffolding,” which is essentially a form of neuroplasticity, enables people to compensate for age-related cognitive decline through the recruitment of alternative brain regions or the generation of new brain cells.

Life-course experiences influence how well someone can “scaffold” (Reuter-Lorenz & Park, 2014). Similar to CR, scaffolding is boosted by lifestyle choices and lifetime experiences, such as education and the engagement in cognitively stimulating activities, which is termed “neural resource enrichment.” Unlike CR, however, STAC-r also considers the effects of adverse life events, such as depression, vascular disease, and head trauma. These events fall under the “neural resource depletion” construct and weaken scaffolding abilities. Aging and neural resource enrichment and depletion contribute to maintaining overall brain status (i.e., the brain’s structure and functional processes). Neural resource enrichment can positively influence the structure and function of the brain, whereas depletion can have a negative influence. Brain structure can be influenced by neuropathology, such as amyloid/tau burden, which would indicate that dementia-related pathology can influence cognitive aging. Brain function can decrease in efficiency (i.e., speed and quality of neural processing) due to changes in, for example, functional brain networks. Brain structure and function are interlinked through the beneficial and adverse influences of neuroplasticity (e.g., neurogenesis and cortical thinning). Moreover, brain structure and function directly contribute to people’s scaffolding abilities.

**Figure 3.**
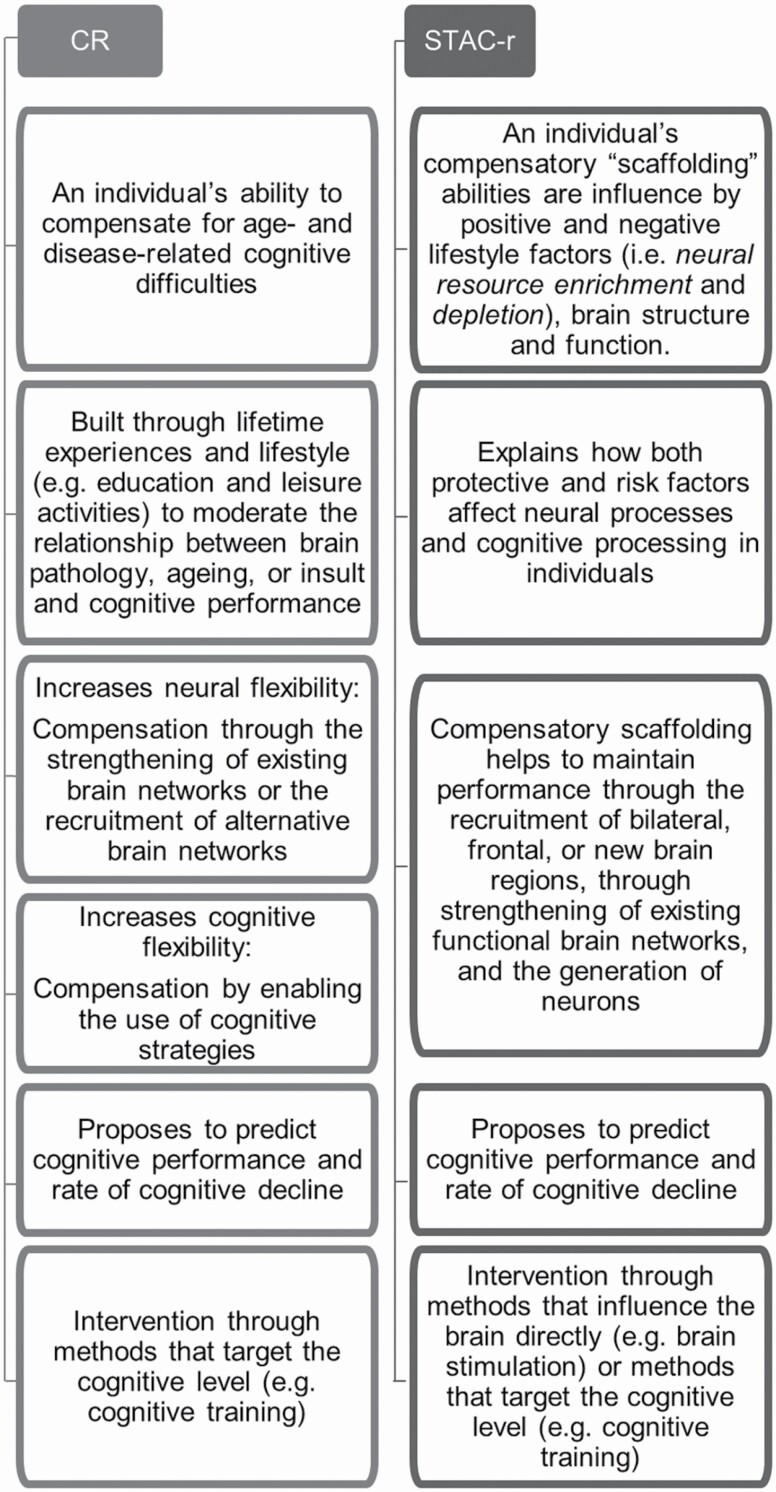
A schematic representation of the main ideas proposed by Cognitive Reserve CR) theory and Scaffolding Theory of Aging and Cognition—revised (STAC-r). Ideas of the two theories about the same theme (e.g., intervention) are aligned to allow for more direct comparison.

In the initial STAC model, the concept of neural dedifferentiation was suggested as a cause of functional deterioration (Park & Reuter-Lorenz, 2009). In the revised STAC model, the concept has been replaced by “neural specificity,” which indicates that neural specificity can decrease with age and through adverse life events but that it can also increase through neural resource enrichment factors (Reuter-Lorenz & Park, 2014). This term corresponds to that of “neural selectivity” proposed by Koen and Rugg (2019). Neural selectivity refers both to reduced brain activity in the brain area underlying a certain cognitive function and to increased brain activity in brain areas that normally do not underly that cognitive function. Furthermore, STAC-r posits that scaffolding ability can improve through intervention, such as through learning and cognitive training as well as through social, physical, and cognitively stimulating activities. The level of cognitive function and the rate of cognitive decline can be predicted through measures of neural resource enrichment and depletion, through observations of brain structure and function, and through compensatory abilities of an individual. Figure 3 compares the proposed mechanisms of STAC-r and CR.

## Overlapping Constructs: The Positive Effects of a Healthy Lifestyle

Both STAC-r and CR agree on the positive effects of a healthy, cognitively stimulating lifestyle. Recent research in healthy older adults demonstrates the positive effects of education and IQ on a wide range of cognitive domains, such as executive functioning, memory, and language (Berggren et al., 2018; Caballero et al., 2021; Lavrencic et al., 2018; Thow et al., 2018). In centenarians, maintaining performance in several cognitive domains was related to higher education and IQ (Beker et al., 2021). Although higher education and IQ might not benefit all cognitive domains equally (Lavrencic et al., 2018; Mungas et al., 2021; Thow et al., 2018), higher education in early life (before the age of 45) has been identified as an important factor in reducing dementia risk in older age (Livingston et al., 2020).

Participation in leisure, social, and cognitively stimulating activities has also been shown to predict cognitive ability in older age (Chan et al., 2018; Grotz et al., 2017). Physical activity, such as aerobic and strength exercise, at different life stages might also improve cognitive functioning in older age (Reas et al., 2019; Sprague et al., 2019) and in centenarians (Beker et al., 2021). A recent meta-analysis indicated that the effect on cognition might depend on the nature of the physical activity (Sprague et al., 2019). For example, aerobic exercise might improve cognitive flexibility, whilst strength training could improve cognitive inhibition. Better cognitive performance in, for example, executive functioning and memory could be due to the generation of nerve cells promoted by physical exercise (Reas et al., 2019).

Because of the positive effects of lifestyle on cognition, both STAC-r and CR propose that the mechanisms compensating for the effects of aging (i.e., scaffolding and reserve) can be enhanced through intervention programs (Klobušiaková et al., 2019; Kocagoncu et al., 2020; Reuter-Lorenz & Park, 2014; Stern, 2013). That is, compensation abilities and reserve can be increased through programs that promote further education and a healthy lifestyle. Furthermore, a healthy lifestyle and interventions, such as cognitive training, can modulate the flexibility of functional brain processes, which is in line with both scaffolding and CR (Reuter-Lorenz & Park, 2014; Stern et al., 2020).

## Where the Theories Part

### STAC-r: Better Capture of the Complexity of Cognitive Aging

Unlike CR, STAC-r incorporates “neural resource depletion,” which includes factors, such as vascular (e.g., smoking) and genetic ones, that negatively influence brain health and compensatory abilities (Reuter-Lorenz & Park, 2014). Brain measures and health factors, such as cortical thinning and high body mass index (BMI), have already been linked to cognitive decline. For example, greater dopamine generation capacity was related to greater cortical thinning in older adults (Ciampa et al., 2021). In this study, cortical thinning was related to decreased working memory performance, but only in people with lower dopamine generation capacity. The relationship between brain function and structure has been demonstrated through studies showing that patterns of functional brain connectivity can change depending on the magnitude and locus of brain tissue deterioration (Vieira et al., 2020), as is predicted by STAC-r (Reuter-Lorenz & Park, 2014). Furthermore, older adults (aged 60–80 years) with similar memory performance to younger adults (aged 18–35 years) exhibit comparable youth-like brain connectivity, indicating the influence of brain function on maintaining cognitive performance (Zhang et al., 2020). However, future studies need to clarify the interplay between neural resource enrichment and depletion factors, intervention, and the bidirectional relationship between brain structure and function.

There is an established relationship between various health measures and cognitive functioning (Caballero et al., 2021; Sebastiani et al., 2020). Specifically, the prevalence of the APOE genotype (i.e., a dementia genetic risk factor), stroke incident, and alcohol abuse predict faster decline of cognitive processing speed, episodic memory (Sebastiani et al., 2020), and executive functioning (Caballero et al., 2021) with age. In centenarians, education and cognitive activities, but not dementia-related brain pathology or genetic risk, were related to cognitive performance (Beker et al., 2021). Hence, compensation mechanisms might stabilize or diverge at very old age, stressing the need for a dynamic model of cognitive aging. STAC-r includes a wider variety of protective and risk factors of cognitive decline, and hence might be more successful than CR in capturing the complex mechanisms involved in cognitive aging.

### Time Course of Compensation Mechanisms

Both STAC-r and CR describe compensation mechanisms (i.e., scaffolding and CR) as dynamic, changing over time due to modulations in overall brain status and in environmental factors, such as lifestyle choices (Reuter-Lorenz & Park, 2014; Stern et al., 2020). STAC-r specifically states that neural resource depletion, enrichment factors, and overall brain status continually contribute to compensation mechanisms, such as neural reorganization and repair (Reuter-Lorenz & Park, 2014). Stern and colleagues (2020) argue that CR is dynamic as lifestyle choices and events change throughout the life span, for example, with education often taking place in early life and professional occupation happening later in life (Stern et al., 2020). However, it is unclear whether CR can be accumulated throughout the life span (Grotz et al., 2017), or whether CR can only be acquired before a certain age (Chan et al., 2018). Finally, CR does not specify whether CR levels decline with age or how long it takes for accumulated CR to increase cognitive and neural flexibility. Hence, future studies should clarify the time course of CR across the life span.

### Rate of Cognitive Decline

The purpose of STAC-r and CR is to predict both age-related cognitive performance and the rate of cognitive decline (Reuter-Lorenz & Park, 2014; Stern, 2013). The association between the proposed constructs of STAC-r and the rate of age-related cognitive decline is supported by several studies. Cognitive decline can be slowed down by factors such as education (Caballero et al., 2021; Sebastiani et al., 2020; but see McKenzie et al., 2020: only in people at genetic risk for dementia), participation in physical activities, and engagement in cognitive activities, such as completing tax forms (Caballero et al., 2021; Reas et al., 2019). However, stroke or cardiovascular risk (Caballero et al., 2021; Sebastiani et al., 2020), dementia genotypes and inflammation (Sebastiani et al., 2020), as well as health risk factors, such as smoking and high BMI (Caballero et al., 2021; Sebastiani et al., 2020), can speed up cognitive decline.

Although CR can help maintain level of cognitive performance, healthy-aging studies do not seem to support that CR slows down cognitive decline (Beker et al., 2021; Berggren et al., 2018; Lavrencic et al., 2018). Moreover, it has been argued that education is not an important proxy for CR in healthy older adults, as it does not predict cognitive change on its own. Rather, brain structure needs also to be considered (Mungas et al., 2021). In contrast, several dementia studies demonstrated slower decline in people with high CR, quantified as the difference between observed and expected (based on structural brain measures and demographics) memory performance, despite the presence of dementia-related pathology (e.g., McKenzie et al., 2020). Warranting further investigation, the effect of CR on the rate of cognitive decline might only become apparent when a certain level of brain pathology is reached.

### CR: Compensation Through Cognitive Strategies

STAC-r proposes that scaffolding happens at a neural level, and that it is stimulated by interventions and cognitive engagement. In contrast, CR posits that compensation through high CR happens at a cognitive level by increasing the efficiency or flexibility of cognitive processes to counter cognitive decline (Stern et al., 2020). However, it is currently unclear what cognitive strategies CR enables. One explanation is that CR allows for faster cognitive processing or that it counteracts the age-related depletion of attentional or working memory resources (Lojo-Seoane et al., 2020). Several aging theories have already proposed that age-related decreases in basic cognitive processes, such as working memory and processing speed, underlie declines in more complex cognitive functions, such as language or decision-making (Hasher & Zacks, 1988; Salthouse, 1990, 1996). That is, poorer performance in older compared to younger adults could be due to slower information processing. However, it could also be caused by a reduction in attentional resources or working memory capacity caused by, for example, difficulties in suppressing irrelevant information. High CR could enhance processing speed, inhibitory control, or attention, which then positively influences other cognitive processes (Lojo-Seoane et al., 2020).

CR can positively influence working memory performance, which then benefits complex cognitive functioning in middle-aged and older adults (Cansino et al., 2020; Lojo-Seoane et al., 2020). That is, there are two explanations for the positive relationship between CR and working memory in older age. Higher CR either leads to better cognition by enhancing working memory directly (Lojo-Seoane et al., 2020), or it serves as an additional resource when working memory resources reach a threshold of depletion (Cansino et al., 2020). Thus, CR could benefit or serve as an additional cognitive resource to the basic cognitive functions described in aging theories, such as theories of limited cognitive resources (Salthouse, 1990). Future research should investigate whether the brain changes that underlie compensatory mechanisms are detectable and what types of neuroplasticity accompany each cognitive change facilitated by CR.

### Compensation at the Level of the Brain

According to STAC-r, to maintain cognitive performance, scaffolding is reflected as an increase in brain activity, recruitment of alternative brain regions, and the generation of neurons (Reuter-Lorenz & Park, 2014). The relationship between brain structure and brain function is well documented (Zahodne et al., 2019). For example, brain structural measures, such as the volume of myelinated content of white matter tracts, predict cognitive processing speed across a wide age range (Chopra et al., 2018). Moreover, compensatory scaffolding can occur in response to brain atrophy by either supporting affected brain areas or recruiting support from less affected brain areas (Vieira et al., 2020). These results indicate that it is possible to characterize brain function without reference to the cognitive level, which is in line with STAC-r.

Stern and colleagues propose that CR ameliorates the effect of brain deterioration on cognition by increasing the efficiency or flexibility of functional brain networks (Stern et al., 2020). That is, brain networks that are more efficient will need less brain activation during cognitive tasks. Flexibility concerns the recruitment of alternative brain networks to maintain cognitive performance. As an example, gray matter loss would have less impact on cognition in people who engage in midlife activities and, hence, have high CR levels (Chan et al., 2018). Recent studies found a positive link between the level of CR and increased efficiency and flexibility of functional brain networks (e.g., Conti et al., 2021). People with low CR measured through educational and occupational attainment, and leisure activity, exhibited similar functional connectivity patterns as was found in a study with people with mild cognitive impairment (MCI; Buldú et al., 2011). Such studies indicate an important role of interventions aimed at increasing CR levels, such as cognitive training, to enable protective mechanisms at the level of functional brain networks.

### STAC-r and CR: Differences in Intervention Methods

STAC-r and CR claim that interventions increase compensatory scaffolding and CR levels, respectively. Intervention programs, such as through brain stimulation or cognitive training, aim at enhancing scaffolding and could improve functional brain processing (Reuter-Lorenz & Park, 2014). Specifically, STAC-r proposes that interventions using brain stimulation could influence brain structure and function directly by enhancing functional connectivity. In line with this proposal, a recent study demonstrated that brain stimulation interventions enabled better cognitive performance in explicit learning, but only in people with low baseline performance (Perceval et al., 2020). That is, brain stimulation could enable compensatory brain mechanisms and improve performance in healthy older adults with suboptimal cognitive performance pre-stimulation. In contrast, executive functioning in older adults might not improve after brain stimulation (Yu et al., 2020). Therefore, future brain stimulation studies might need to consider baseline cognitive performance to detect any beneficial effects of stimulation on cognitive performance.

In addition to brain stimulation, STAC-r proposes that interventions at a cognitive level, such as cognitive training and engagement in cognitive and physical activities, can increase compensatory scaffolding abilities (Reuter-Lorenz & Park, 2014). Brain structure and function could also directly influence compensatory scaffolding abilities (Reuter-Lorenz & Park, 2014). Indeed, the strength of functional connectivity can predict the outcomes of cognitive training. That is, stronger functional connectivity leads to higher training gains in certain cognitive functions (Faraza et al., 2021). Hence, the effect of intervention on compensatory scaffolding could be dependent on brain status. In contrast, CR proposes that interventions should happen at a cognitive level (Stern, 2013). That is, interventions are based on proposed CR proxies, such as cognitive or physical activity, and do not directly increase neuroplasticity through brain stimulation. Such interventions then lead to changes which can be observed both at the cognitive and the neuronal level. For instance, cognitive training, such as learning new skills, was shown to enhance functional connectivity (van Balkom et al., 2020), in line with both STAC-r and CR.

## Relevance of STAC-r and CR to Neurodegenerative Diseases

STAC-r is primarily concerned with age-related cognitive decline but could also be applied to neurodegenerative diseases. The role of STAC-r in neurodegenerative disease is indicated by the influence of neuropathology (e.g., amyloid/tau burden), on brain structure (Reuter-Lorenz & Park, 2014). Factors such as stroke incident, years of education, and brain atrophy could be used as predictors for the risk of and transition into MCI or neurodegenerative diseases (Xu et al., 2020). Furthermore, the relationship between deterioration of brain structure, changes in functional brain networks, and subsequent cognitive decline has been demonstrated in dementia (Klobušiaková et al., 2019; Kocagoncu et al., 2020). For example, tau burden (i.e., that of proteins in nerve cells) in Alzheimer’s disease is associated with changes in functional brain networks, which can lead to the worsening of cognitive functions (Kocagoncu et al., 2020). The efficiency of functional brain networks as measured through resting-state functional neuroimaging analyses has been linked to MCI and neurodegenerative diseases (Klobušiaková et al., 2019). These studies show that, compared to healthy adults, people with MCI, Alzheimer’s disease, or Parkinson’s disease show a reduction in brain efficiency, which correlates with the severity of cognitive impairments. In Parkinson’s disease specifically, reductions in brain efficiency may indicate pre-onset cognitive dysfunction (Klobušiaková et al., 2019). Post-onset cognitive dysfunction in people with Parkinson’s disease with MCI might be related to increased brain efficiency, potentially indicating a compensatory mechanism for their cognitive impairment in response to changes in brain status.

High CR can delay the onset of dementia (Stern, 2013; Stern et al., 2020; Xu et al., 2020) and lessen the impact of this disease on cognitive functioning (Stern et al., 2020). At later stages of neurodegenerative diseases, CR might not be enough to compensate for the negative impact of brain pathology (Stern et al., 2020). Consequently, there is an acceleration in the rate of cognitive decline (Zahodne et al., 2019). CR could decrease the negative impact on cognition caused by age-related brain pathology, such as white matter hyperintensities (Zahodne et al., 2019), and might delay the onset of dementia symptoms by optimizing functional brain networks and increasing the brain’s efficiency of information transfer (Li et al., 2021).

Taken together, both STAC-r and CR could be useful in predicting dementia risk, onset, and disease progression. Although STAC-r has not often been discussed specifically in light of neurodegenerative diseases, STAC-r could lead to more accurate predictions as it considers both the positive effects of lifestyle and the influence of adverse health factors. How the different components of STAC-r interact with each other to explain disease-related cognitive impairments warrants further research. Finally, the protective effects of CR might be reduced when someone is at a genetic risk for dementia (Li et al., 2021). Alternatively, CR might mitigate genetic risk factors of dementia (Beker et al., 2021). These findings stress the importance of a comprehensive model that also considers adverse health factors.

## Concluding Remarks and Future Perspectives

In this review, we have discussed two frameworks that can explain the mechanisms that compensate for cognitive decline in healthy aging and neurodegenerative diseases. Both scaffolding and CR involve increased neural efficiency, and the ability to scaffold or compensate can be influenced by lifestyle factors and interventions. While CR provides a framework for the effects of an enriching lifestyle on late-life cognition, STAC-r offers a more multifaceted approach that better encompasses the complex mechanisms of age- and dementia-related cognitive decline. Recent research supports the interaction of neural resource enrichment and depletion (e.g., healthy lifestyle and APOE genotype respectively; Caballero et al., 2021; Sebastiani et al., 2020). Hence, future studies would benefit from considering not only healthy lifestyle factors, but also factors that negatively influence cognitive aging. Indeed, researchers have argued that both models could be combined in that resilience (i.e., cognitive reserve, and brain reserve and maintenance) might contribute to compensatory mechanisms to maintain cognition (Cabeza et al., 2018).

Both STAC-r and CR suffer from shortcomings in their explanations of age- and disease-related cognitive decline (Table 2). Hence, future studies should aim to address the weaknesses of both theories in pursuing evidence-based intervention and prevention strategies against dementia. Neurodegenerative diseases exhibit distinct profiles, including disease-related changes of functional brain networks (Klobušiaková et al., 2019). STAC-r could potentially be developed into disease-specific submodels for generating prognoses of cognitive decline, which could serve as a tool for predicting the onset and progression of dementia. Regarding CR, it is important to clarify the time window for building CR, so that intervention programs can be implemented at the right age. Finally, more research is needed to understand the importance of different CR proxies and their influence on different cognitive domains to determine what combination of proxies should be targeted in interventions.

**Table 2.** Identified Strengths and Shortcomings of STAC-r and CR

Shortcomings	Strengths
STAC-r • The role of STAC-r in neurodegenerative diseases is underspecified	• STAC-r might be able to predict the onset and rate of cognitive decline brought on by neurodegenerative diseases (see, e.g., Kocagoncu et al., 2020)
	• STAC-r might be more suitable for predicting the rate of cognitive decline as opposed to CR
	• STAC-r proposes a specific link between brain processes and compensation/resilience through scaffolding
CR	
• CR does not clearly explain the relationship between cognitive, brain, and functional reserve, making it difficult to test the theory	• In contrast to STAC-r, CR offers a simpler framework that might be easier to use for designing focused intervention programs
• No neural basis for CR has been proposed. Hence, it is unclear what specifically underlies CR	• CR contributes to understanding the onset and progression of dementia symptoms. Therefore, CR might better apply to cognitive decline in dementia than STAC-r
• There is still no consensus on whether each CR proxy weighs equally when estimating CR (Borgeest et al., 2020) or whether some proxies, such as education, are more important than others, such as engaging in leisure activities (Chan et al., 2018; Grotz et al., 2017)	
• It is currently unclear whether CR can be accumulated throughout the life span (Grotz et al., 2017), as proposed by CR (Stern et al., 2020), or whether there is a “critical period” for building CR (Chan et al., 2018), for example, before very old age (i.e., the age of 100 years; Beker et al., 2021)	
• CR proxies can predict cognitive performance, but the rate of decline is altered by factors other than CR proxies. Predicting the rate of cognitive decline is paramount for identifying detrimental cognitive problems at an early stage	

*Note*: CR = Cognitive reserve; STAC-r = Strengths of the Scaffolding Theory of Aging and Cognition—revised.

As the mean age of the global world population increases rapidly and dementia becomes more prevalent in our societies, it is of paramount importance to develop our understanding of the mechanisms that allow for healthy cognitive aging. Therefore, we need to develop a better understanding of the mechanisms that compensate for dementia-related cognitive decline and slows the onset of the disease. Both STAC-r and CR provide promising explanations for cognitive decline with age and in neurodegenerative diseases. They both identify a range of factors that predict healthy cognitive aging, and STAC-r also specifies factors that predict the rate of cognitive decline. It is likely that these predictive qualities can be boosted using machine-learning algorithms, which could provide a set of tools that would allow for early intervention, improved compensatory abilities, and delay in the onset of dementia symptoms.
